# Anatomical observation and transcriptome analysis of buds reveal the association between the AP2 gene family and reproductive induction in hybrid larch (*Larix kaempferi × Larix olgensis*)

**DOI:** 10.1093/treephys/tpac111

**Published:** 2022-09-23

**Authors:** Jun-Fei Hao, Chen Wang, Chen-Rui Gu, Dai-Xi Xu, Lei Zhang, Han-Guo Zhang

**Affiliations:** State Key Laboratory of Tree Genetics and Breeding, Northeast Forestry University, No. 51 Hexing Road, Xiangfang District, Harbin 150040, China; State Key Laboratory of Tree Genetics and Breeding, Northeast Forestry University, No. 51 Hexing Road, Xiangfang District, Harbin 150040, China; State Key Laboratory of Tree Genetics and Breeding, Northeast Forestry University, No. 51 Hexing Road, Xiangfang District, Harbin 150040, China; State Key Laboratory of Tree Genetics and Breeding, Northeast Forestry University, No. 51 Hexing Road, Xiangfang District, Harbin 150040, China; State Key Laboratory of Tree Genetics and Breeding, Northeast Forestry University, No. 51 Hexing Road, Xiangfang District, Harbin 150040, China; State Key Laboratory of Tree Genetics and Breeding, Northeast Forestry University, No. 51 Hexing Road, Xiangfang District, Harbin 150040, China

**Keywords:** anatomical observation, endogenous hormone, floral induction, gymnosperms, strobilus, transcriptome

## Abstract

Hybrid larch is an excellent afforestation species in northern China. The instability of seed yield is an urgent problem to be solved. The biological characteristics related to seed setting in larch are different from those in angiosperms and other gymnosperms. Studying the developmental mechanism of the larch sporophyll can deepen our understanding of conifer reproductive development and help to ensure an adequate supply of seeds in the seed orchard. The results showed that the formation of microstrobilus primordia in hybrid larch could be observed in anatomical sections collected in the middle of July. The contents of endogenous gibberellin 3 (GA3) and abscisic acid (ABA) were higher and the contents of GA4, GA7, jasmonic acid and salicylic acid were lower in multiseeded larch. Transcriptome analysis showed that transcription factors were significantly enriched in the AP2 family. There were 23 differentially expressed genes in the buds of the multiseeded and less-seeded types, and the expression of most of these genes was higher in the buds than in the needles. We conclude that mid-July is the early stage of reproductive organ development in hybrid larch and is suitable for the study of reproductive development. GA3 and ABA may be helpful for improving seed setting in larch, and 23 AP2/EREBP family genes are involved in the regulation of reproductive development in larch.

## Introduction

Larch (*Larix* spp.) is the main afforestation species in northern China. It grows rapidly and has a straight stem form. Larch is a commercial timber used in construction and plays an important role in civil and strategic reserves (Jia and Zhou 2018). Since the provenance experiment in the 1980s, China has been carrying out excellent variety selection and cross breeding. In the process of genetic improvement for >40 years, it was found that the local larch (*Larix olgensis* Henry) and the introduced larch (*Larix kaempferi* (Lamb.) Carr.) had obvious heterosis during growth (Han et al. 2014). Hybrid larch (*L. kaempferi × L. olgensis*) is the main material for the establishment of high-generation larch seed orchards at present. Because the tissue culture system of conifers has been very difficult to establish, larch seed orchards provide excellent reproductive materials via the stable productions of seeds for afforestation (Tretyakova et al. 2018). However, the seed setting cycle of larch is long, and the seed yield is unstable. Studying the biological characteristics and development process of sporophylls will help us to further understand the reproductive mechanism of larch.

Plant flowering generally requires two important stages: floral induction and flower organ development. Floral induction involves multiple pathways: the photoperiodic pathway (Wang et al. 2021), vernalization pathway (Henderson et al. 2003), gibberellin (GA)-signalling pathway (Dong et al. 2017) and autonomous pathway (Cheng et al. 2017). These studies have shown that in the process of floral induction, plants need to first sense various environmental signals, such as light (Meng and Runkle 2019) and temperature (Verhage et al. 2014), combine them with the internal state (Simpson 2004) and finally summarize the signal via floral integrators, such as FLOWERING LOCUS T (*FT*) (Lee et al. 2013) and SUPPRESSOR OF OVEREXPRESSION OF CO 1 (*SOC1*) (Moon et al. 2003). These integrators go on to activate flower meristem determining genes, such as APETALA 1 (*AP1*) and LEAFY (*LFY*) (Goslin et al. 2017), to complete induction and enter the flowering determination state. Then, the plant enters the stage of flower organ development. Plants need the sequential expression of a series of floral organ identity genes (such as SQUA/*AP1*, APETALA 3 (*AP3*)/PISTILLATA (*PI*) (Wuest et al. 2012) and AGAMOUS (*AG*) (Pelayo et al. 2021)) to complete this stage. These genes have been summarized into the ABC(DE) model, in which all genes belong to the MADS-box family, except APETALA 2 (*AP2*) (Yant et al. 2009). Larch is monoecious and belongs to the gymnosperms, which have no true flowers but use the sporophyll as the reproductive organ (Groth et al. 2011). Here, we use the phrase floral induction to express the induction stage of the early reproductive development of hybrid larch. The sporophylls differentiate in the first year and winter in bud scales, where they continue to develop and open in the spring of the second year, so it is difficult to distinguish the developmental stage of the sporophylls in the early stage. At the same time, unlike *Pinus koraiensis*, *Pinus tabulaeformis* and other *Pinus* species, which have megasporophylls attached to the ends of branches at the top of the canopy, larch megasporophylls and microsporophylls are distributed throughout the canopy (Nguyen et al. 2018). Due to the alternate bearing phenomenon, it is difficult to study the reproductive development of larch.

Through anatomical observation, endogenous hormone determination and transcriptome sequencing, we studied the differences between the two seed–setting types at the end of the floral induction stage to identify the genes related to hybrid larch floral induction. This study provides a valuable reference for the study of genes related to sporophyte development in gymnosperms and may help to improve seed yield in the process of genetic improvement of larch.

## Materials and methods

### Source of test materials

In the hybrid larch seed orchard (20 years old), which reached the age of general seed setting, we selected two kinds of hybrid larch that produced more seeds (DH1, DH2, Clone No. 96-47) and almost no seeds (CK1, CK2, Clone No. 97-112) with the same site conditions, and we collected new buds on the 2- to 4-year-old branches in the middle and lower tree canopy. The seed orchard adopts the cluster configuration, and there are four trees in each cluster, including two clones for each of two varieties, with a row spacing of 4 m × 4 m, and 6 m × 6 m between clusters. The stand is located in Linkou County, Mudanjiang City, China (4 45°24′45. 49″ N, 130°32′55. 10″ E). The anatomical observation samples were collected on 30 June, 15 July and 30 July. The samples were then soaked in FAA and stored at 4 °C. Flowering is greatly affected by the environment. In order to ensure the consistency of environmental conditions, two biological repeats in the same cluster were set for each genotype for transcriptome sequencing. Two biological replicates and three technical replicates were set for each genotype to determine endogenous hormones. The sampling period for hormone determination and transcriptome sequencing was July 20. After collection, the samples were quickly put into liquid nitrogen for storage.

### Anatomical observation

The new buds of hybrid larch collected in June 30, July 15 and July 30 were soaked in formaldehyde-acetic acid-ethanol (FAA) fixation solution for 1 week, dehydrated with gradient alcohol, cleared with xylene and embedded in paraffin. Then, 8-μm longitudinal sections were cut on a paraffin slicer (HM340E, Thermo, Waltham, MA, USA). After safranine solid green staining, bud development was observed under an optical microscope (Axio, Zeiss, Oberkochen, Germany), and photographs were taken.

### Determination of endogenous hormones

The endogenous hormones in the materials were extracted with isopropanol water hydrochloric acid, and the contents of indoleacetic acid, abscisic acid (ABA), CIS zeatin riboside, trans-zeatin-riboside (TZR), dihydrozeatin riboside (DZR), jasmonic acid (JA), salicylic acid (SA), GA3, GA4 and GA7 in the materials were determined by liquid chromatography (Agilent 1290, Agilent, USA) and tandem mass spectrometry (AB SCIEX-6500Qtrap, AB, USA).

### Transcriptome sequencing and quality control

A total of 1 μg of RNA per sample was used as input material for the RNA sample preparations. Sequencing libraries were generated using the NEBNext®Ultra™ RNA Library Prep Kit for Illumina® (NEB, USA) following the manufacturer’s recommendations, and index codes were added to attribute sequences to each sample. Fragmentation was carried out using divalent cations under elevated temperature in NEBNext First-Strand Synthesis Reaction Buffer (5X). First-strand cDNA was synthesized using random hexamer primers and M-MuLV Reverse Transcriptase. Second-strand cDNA synthesis was subsequently performed using DNA Polymerase I and RNase H. The remaining overhangs were converted into blunt ends via exonuclease/polymerase activities. After adenylation of the 3′ ends of DNA fragments, NEBNext Adaptors with hairpin loop structures were ligated to prepare for hybridization. Library fragments were purified using the Ampure XP system (Beckman Coulter, Beverly, USA). Then, 3 μl of USER Enzyme (NEB, USA) was combined with size-selected, adaptor-ligated cDNA at 37 °C for 15 min followed by 5 min at 95 °C before PCR. PCR was performed with Phusion High-Fidelity DNA polymerase, Universal PCR primers and Index (X) primer. Finally, PCR products were purified (AMPure XP system), and library quality was assessed on the Agilent Bioanalyzer 2100 system.

The clustering of the index-coded samples was performed on a cBot Cluster Generation System using TruSeq PE Cluster Kit v3-cBot-HS (Illumina) according to the manufacturer’s instructions. After cluster generation, the library preparations were sequenced on an Illumina HiSeq 2000 platform, and paired-end reads were generated. All high-throughput transcriptome data have been uploaded to CNCB database (cncb.ac.cn) under accession number: CRA007533. The quality of the filtered data was controlled by FastQC (v0.11.7) and spliced with Trinity (v2.0.6), and then the contigs were assembled into transcripts. Gene function was annotated based on the following databases: NR (NCBI nonredundant protein sequences), Pfam (Protein family), Swiss-Prot (a manually annotated and reviewed protein sequence database), Kyoto Encyclopedia of Genes and Genomes (KEGG) and Gene Ontology (GO).

### Analysis of differentially expressed genes

The differentially expressed genes (DEGs) were analysed by DESeq2 for GO enrichment and KEGG enrichment. The enriched gene families annotated in Pfam were analysed by the chi-square test. After obtaining the protein sequences of the Arabidopsis AP2/EREBP family from TAIR (www.arabidopsis.org), the phylogenetic tree of this gene family in hybrid larch was constructed by the neighbour-joining method. After normalizing the FPKM values (Z score), we drew an expression heatmap.

### qRT–PCR analysis

Primer pairs were designed for each gene using Primer 6.0 software (Table S1 available as Supplementary data at *Tree Physiology* Online). Ten DEGs were randomly selected for reverse transcription and quantitative PCR (qRT–PCR) analysis to verify the reliability of the RNA-seq analysis. RNA was extracted from buds using a PureLink™ Plant RNA Kit (Takara, Dalian, China). Reverse transcription was performed using a PrimeScript RT Perfect Real Time Kit (Takara). α-Tubulin was chosen as a reference gene. The mixed materials of CK1B and CK2B were used as controls. The fluorescence quantitative reagent was from the SYBR Premix Ex TaqII (Tli RNaseH Plus) Kit (Takara). qRT–PCR was carried out with a qTOWER 3G cycler. The relative expression of transcripts was determined by the 2^−ΔΔCT^ method. Each sample was repeated three times.

## Results

### Observation of the anatomical morphology of buds at the physiological differentiation stage

**Figure 1.**
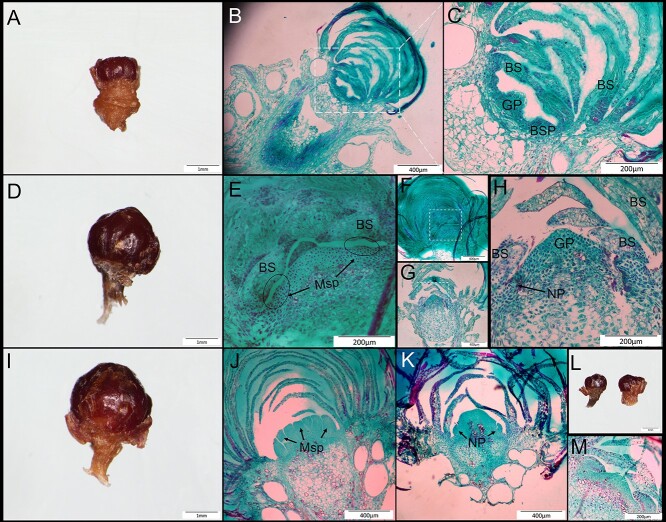
Longitudinal anatomical structure of hybrid larch buds. (A) The end stage of bud scale differentiation (30 June). (B) Longitudinal anatomical structure of bud in late June. (C) Partial magnification of B. (D) The initial stage of microstrobilus primordium formation (15 July). (E) Partial magnification of F. (F) Longitudinal anatomical structure of microstrobilus bud in the middle of July. (G) Longitudinal anatomical structure of ovulate strobilus bud in middle July. (H) Partial magnification of G. (I) Early stage of sexual organ development (30 July). (J) Longitudinal anatomical structure of microstrobilus bud in late July. (K) Longitudinal anatomical structure of ovulate strobilus bud in late July. (L) Bud of needle leaves in middle July (left) and late July (right). (M) Anatomical structure of needle leaves bud in late July. GP, growing point; BS, bud scale; BSP, bud scale primordium; Msp, microsporophyll primordium; NP, needle primordium.

There is a period of physiological differentiation before the completion of floral induction. Because larch buds are very small and wrapped in bud scales, it is difficult to distinguish the types of buds from the external morphology in the early stage of development as it can only be observed by anatomical methods. In the early stage of larch bud development, the apical meristem forms bud scales and then continues organ development from the growing point. The anatomical structure shows that in late June (30 June), the bud is at the end of bud scale formation (Figure 1). At this time, most bud scales have been differentiated, and the growing point is only a small protrusion. There is an interval of ~100 μm between the bud scale primordium and the growing point. It is not yet possible to distinguish leaf buds from spore bulb buds based on external morphology or anatomical observation. By the middle of July (July 15), the shape of reproductive buds was slightly larger than that of leaf buds, but it was not obvious. The anatomical structure is clearly distinguished at this stage. The tip of the growing point of microstrobilus develops into a flat shape, with slight protuberance on the edge, which is the primordium of the microsporophyll. The growing point apex of the ovulate strobilus is sharp. There are two or three layers of basal needles at the bottom of the mature ovulate strobilus, which means that the ovulate strobilus and the needles bud have the same needle primordium structure in the early stage of development. In addition, the size of ovulate strobilus and needles bud are similar in the middle of July, so it is difficult to distinguish them at this time. Newly appeared microsporophyll primordium indicates that this period is the early stage of larch flower organ development (the end of floral induction). By the end of July, the female strobilus (Figure 1K) has obvious needle primordia at this time. What distinguishes it from leaf buds (Figure 1M) is that the apex is obviously uplifted. Meanwhile numerous obvious microsporophyll primordia appeared in microstrobilus (Figure 1J). It shows that late July is the early stage of sexual organ development. Therefore, in order to find out the key factors affecting the flowering induction of larch, we should study the buds between middle and late July.

### Determination of endogenous hormones in hybrid larch at the end stage of floral induction

**Figure 2.**
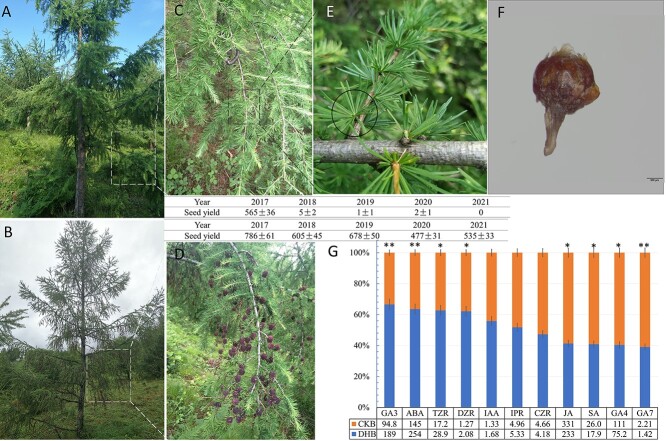
Twenty-year-old hybrid larch seed orchard (mid- July 2019). (A) Larch that hardly bears seeds in lean years. (B) Larch that maintains stable seed set in lean years. (C) Branches that hardly bear seeds. (D) Normally seeded branches. (E) Dwarf shoots of larch (bud at the centre). (F) New buds with bud scales (these will unfold into a needle or strobilus in the second year), scale: 500 μm. (G) The content (ng g^−1^) and proportion of endogenous hormones in the buds of two seed setting types of larch.

Larch seed setting undergoes a common phenomenon of alternate bearing, with 5–7 years being the harvest cycle (Smith and Samach 2013). Most larch trees have little or no seed setting in successive lean years, but some plants maintain stable seed setting in successive years (Figure 2), which provides enough material for studying the reproductive mechanism of larch. According to its anatomy, the endogenous hormones of two types of larch with similar microsite and light conditions were determined in the middle of July (Figure 2G). The results showed that at the end stage of floral induction, the contents of GA3 and ABA in the buds of multiseeded trees (DHB) were significantly higher than those of less-seeded trees (CKB). The contents of TZR and DZR in DHB were also high, ~1.6 times that of CKB. The contents of GA4 and GA7 in DHB were significantly lower than those in CKB, and the contents of JA and SA, which are closely related to resistance, were also significantly lower than those in CKB.

### Transcriptome sequencing and DEG analysis of two seed setting types of hybrid larch buds

To determine the difference in mRNA expression between the buds of the two seed setting types of hybrid larch at the end stage of floral induction, the buds and their surrounding needles were collected and subjected to high-throughput sequencing; a total of 25.5 GB of clean data were obtained. A total of 159,991 transcripts (*N*50 = 1840 bp) and 100,141 unigenes (*N*50 = 1341 bp) were obtained by combined assembly. Among them, 21231278 (buds of multiseeded tree 1, DHB1), 22030256 (buds of multiseeded tree 2, DHB2), 27135100 (buds of less-seeded tree 1, CKB1) and 27216256 (buds of less-seeded tree 2, CKB2) reads were obtained for each library. The ratio of reads compared with transcripts or unigenes was >80%.

**Figure 3.**
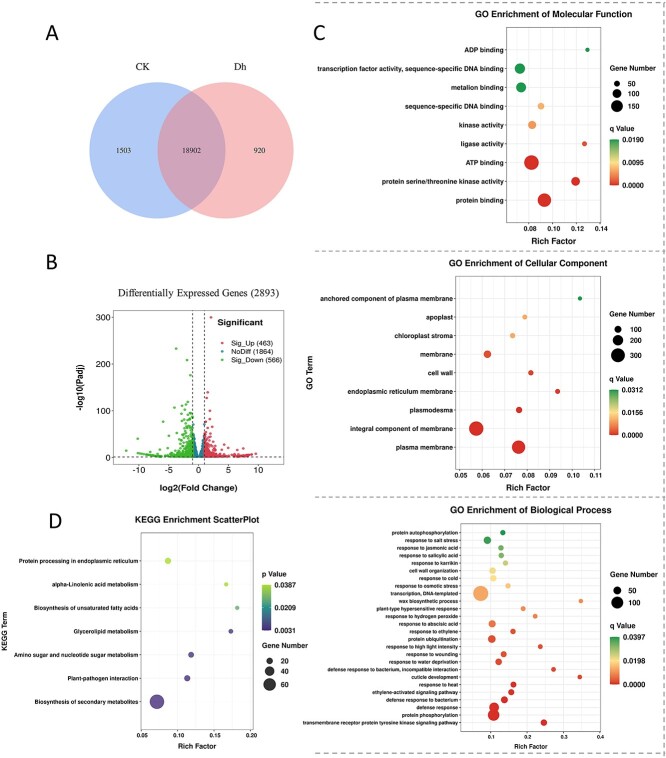
Analysis of DEGs in the buds of two seed setting types of hybrid larch. (A) Venn diagram of the number of genes identified between the two groups of materials. (B) Volcano plots of DEGs; the significance level was set as |log2FC| > 2, Padj (FDR) < 0.05. (C) GO term enrichment analysis of differential genes; *q* value (FDR) < 0.05. (D) KEGG enrichment analysis of DEGs, *P* value < 0.05.

According to the criteria of false discovery rate (FDR) <0.01 and fold change >2, there were 2893 DEGs in the two groups of buds (Figure 3), of which 463 were significantly upregulated and 566 were significantly downregulated in DHB. The results of GO term enrichment of DEGs showed that in terms of molecular functions, DEGs were mainly enriched in the binding of various proteins (GO:0004674 protein serine/threonine kinase activity, GO:0005515 protein binding), the activity of transcription factors (TFs; GO:0003700 TF activity, sequence-specific DNA binding) and the operation of energy (GO:0005524 ATP binding, GO:0043531 ADP binding). In terms of cell components, DEGs were mainly enriched in the cell wall, plasmodesma and various membrane structures. In terms of biological processes, DEGs were mainly enriched in response to various environmental signals (response to heat, cold, osmotic stress, water deprivation, salt stress, high light intensity, wounding, etc.), phytohormone signals (response to ethylene, ABA, SA, JA) and transmembrane proteins related to signal transduction. Therefore, it can be speculated that in hybrid larch, trees receive signals from the external environment, activate TFs through a series of signal transduction cascades and then promote the differentiation of sporophylls. During this period, plant hormones such as ethylene and ABA are involved as regulatory molecules.

### Analysis of differentially expressed TF families

**Figure 4.**
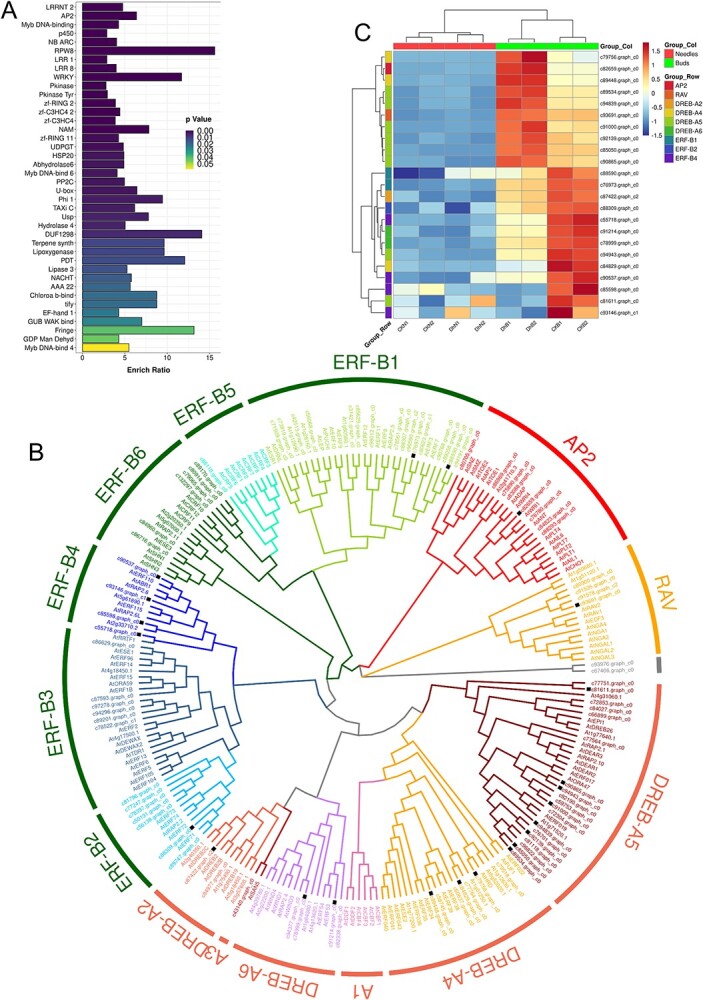
(A) Gene family enrichment analysis of DEGs; *P*-value (Bonferroni) <0.05. (B) Phylogenetic analysis was performed using the neighbour-joining (NJ) method. Eighty-seven genes in *L. kaempferi* × *L. olgensis* were divided into 13 subfamilies of the AP2/EREBP family, where black squares indicate DEGs. (C) Heatmap of AP2/EREBP gene expression in needles and buds of hybrid larch in this period.

Transcription factors participate widely in the regulation of gene expression during plant development. The same subfamily usually has similar or complementary functions (Dreni and Kater 2014). Through the statistical and enrichment analysis of the families of DEGs annotated in the Pfam database (Figure 4A), the TF families that were significantly enriched included AP2, Myb DNA-binding and WRKY. The most significantly family enriched was the AP2 family. A total of 87 genes from the AP2/EREBP family were found in the transcripts and unigene library of hybrid larch during this period (Figure 4B). The phylogenetic tree showed that these 87 genes were distributed across 14 AP2/EREBP subfamilies, except the DREB-A1 subfamily, and two genes were not classified into any subfamily. A total of 23 of these AP2/EREBP genes were differentially expressed in the two groups of materials. Among them, 10 were upregulated in DHB, and 13 were downregulated. The AP2, RAV and DREB-A2 subfamilies have only one DEG, and the DREB-A6 subfamily has only two DEGs, which were both upregulated in DHB. There were three DEGs in the DREB-A4 subfamily, two of which were upregulated in DHB and the other of which was downregulated. The DREB-A5 subfamily has eight DEGs, six of which were upregulated and two of which were downregulated in DHB. All DEGs of the ERF subfamily (ERF-B1, ERF-B2 and ERF-B4) were downregulated in DHB. Genes related to reproductive development are usually expressed in buds during differentiation. To confirm this hypothesis, transcriptome sequencing was performed on needles near the sampling location of the buds. The expression heatmap (Figure 4C) showed that the expression of most of these 23 genes was significantly higher in buds than in needles.

### Verification of the transcriptome by real-time qRT–PCR

**Figure 5.**
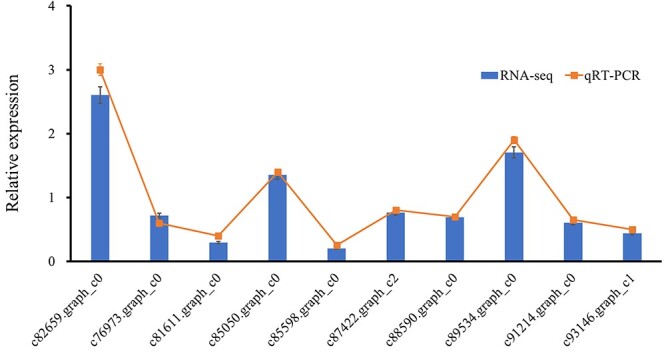
The relative expression levels of 10 DEGs based on RNA-seq and qRT–PCR. The relative gene expression levels were determined by the 2^−ΔΔCT^ method.

To verify the difference between the libraries produced for the two groups of materials, we randomly selected 10 differentially expressed AP2/EREBP genes from hybrid larch and performed qRT–PCR analysis (Figure 5). The relative expression of these genes is highly consistent with the results of RNA-seq, indicating the reliability of transcriptome data analysis.

## Discussion

### Limitations of the development period and materials

Generally, the reproductive development of adult plants requires a floral induction stage and an organ development stage (Hempel et al. 2000). In the floral induction stage, the apical meristem differentiates into reproductive organs after receiving environmental and endogenous signals. The organ development stage occurs when the buds differentiate into various parts of reproductive organs after induction. Problems in these two stages can lead to a reduction in flowering. The difference is that the induction stage can determine the number of flower buds, which is mainly affected by the environment. Abnormalities in the organ development stage may lead to seed abortion (Smith and Zhao 2016), which is greatly affected by genes within the plant. The development period of larch buds is long. It takes almost 9 months from the formation of the new bud to the completion of insemination, and for >7 months, the seed is wrapped under the bud scale. Based on observational data, the key period of bud differentiation also occurs in these 7 months, which makes it difficult to distinguish the type (needle or strobilus) and gender of buds based on morphology. However, measured after anatomical observation, endogenous hormones and mRNA can very easily degrade. Therefore, we can only determine the different seed setting plant types in consecutive years based on statistics. Because the number of male/female sporophylls at the time of sampling cannot be known until the blossom of the second year, the time span of this research is large. Thus, we observed the seed setting for 3 years to determine the beginning of continuous years and to identify different seed setting types of plants, resulting in a 1-year sampling period (buds taken in 2019 will bloom in 2020), and 2-year investigation and verification period. This long timeframe makes the results more reliable.

The GO enrichment results of the transcriptome showed that terms related to the response to the environment, signal transduction and TF activity were enriched. The characteristics of the sampling period were consistent with those at the end of floral induction, which confirmed the conclusions based on anatomical observations. However, the key factor affecting the seed setting yield of larch is the number of ovulate strobilus. Although the materials used to analyse DEGs came from two groups of trees with great differences in seed setting, it was difficult to estimate how much the ‘proportion of female buds’ has affected the difference. Therefore, if we can determine the sex of reproductive buds and then analyse the differential genes, we can find out the genes that affect the differentiation of ovulate strobilus and microstrobilus, respectively, so as to be more targeted for the study of promoting seed setting. This idea faces two problems: (i) at the end of April in the second year, male and female cones can be distinguished, but the time is nearly 9 months from the beginning of differentiation. There is great uncertainty whether the results can represent the situation at that time. (ii) In addition to the anatomical structure, it is difficult to identify the sex of reproductive buds in the early stage. The long-term fixation and embedding during the operation will inevitably lead to the degradation of mRNA. Imitating the method of freezing microtomy used before spatial transcriptome sequencing (Liu et al. 2022), although it can prevent RNA degradation to a certain extent, the thick seed scales make it difficult to maintain a clear structure during rapid freezing and fixation. These two points are also part of the research that our laboratory is currently engaged in.

### Phytohormones and reproductive development

**Figure 6.**
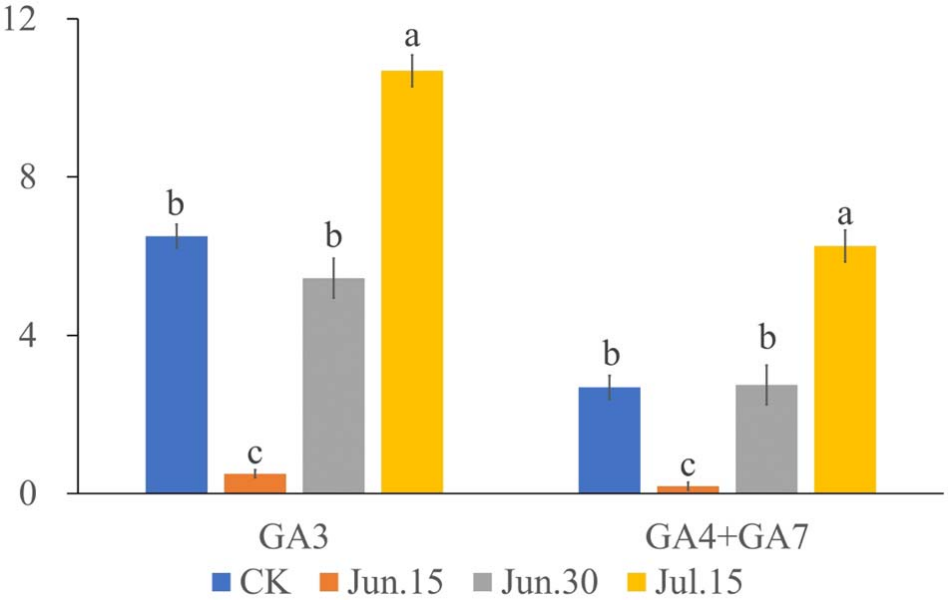
Effect of hormone treatment time on seed setting of hybrid larch. Hormone concentration 500 mg l^−1^, dose 2 ml. Each kind of hormone treatment test includes 64 trees, of which 16 trees are used as control, and 16 trees are treated at each time, a total of three times (15 June, 30 June, 15 July). GA3 treats four plants of each clone number: No. 85, No. 127, No. 281 and No. 320 each time. GA4 + GA7 treats four plants of each clone number: No. 40, No. 56, No. 283 and No. 592 each time. Plot with the average value of each treatment.

Phytohormones play an important role in all processes of plant growth and development. Studies have shown that hormones are involved in many stages of flowering (Iqbal et al. 2017). GA plays an important role in flower induction and organ development (Li et al. 2018). In the floral induction period, the content of GA3 in plants with more seeds was significantly higher than that in plants without seeds, which indicates that GA3 may play a certain role in the flowering induction of hybrid larch (Russell and Hak 2007). GA4 and GA7 showed the opposite results. According to a previous report (Almqvist 2003), it is speculated that GA4 and GA7 may play a role in organ development rather than induction. At the same time as the above experiment, we explored the effect of treatment time on the seed setting of hybrid larch promoted by two GAs through stem injection (Figure 6). Due to the low-seed setting rate in the lean year, the data are not ideal, so it is not listed in the Results section, but only here for reference. It can be seen that the treatment time does affect the effect of GA on stimulating larch seed setting to a certain extent. In addition, there is also a high content of ABA in the multiseeded buds. Some studies say that ABA promotes flowering by positively regulating the expression of *FT* and TWIN SISTER OF FT (*TSF*) (Riboni et al. 2013), but others show that ABA inhibits flowering (Wang et al. 2013). Further research is needed on the specific role of ABA in the flowering process of larch. It is worth noting that the contents of JA and SA are significantly lower in multiseeded hybrid larch, and these two kinds of hormones are mainly related to the plant defence response (Zhang and Li 2019, Ali and Baek 2020, Ding and Ding 2020). Studies have shown that certain stresses can promote flowering (Wada et al. 2010). Functional studies of some genes have also identified genes that can enhance resistance and lead to delayed or reduced flowering, such as *AtDDF1* (Magome et al. 2004) and *AtSAN5*(Shu et al. 2016). Therefore, it is speculated that there is a certain correlation between the number of female cones and resistance. However, whether the high content of endogenous JA and SA is a manifestation of high resistance or a stress response due to low resistance remains to be studied. Most of the AP2/EREBP family genes in this study are involved in the biosynthesis and signal transduction of various hormones. In future research, clarifying the upstream and downstream relationships between hormones and TFs can provide some guidance for the regulation of larch reproduction by external hormone application.

### MADS box and AP2 at the development stage

In the ABC(DE) model of plant floral organ development, there are only two kinds of key genes. Except for APETALA2 (*AP2*), the other genes belong to the MADS-box family (Yant et al. 2009). However, some DEGs belonging to the MADS-box family were not significantly enriched. This is because MADS-box genes are mainly involved in the development of flower organs, and our sampling period was at the end of flower induction (early organ development). In this period, more environmental signals are pooled and combined with floral meristem identity genes (Litt 2007); thus, the AP2/EREBP family is more enriched.

APETALA2 (*AP2*) in the AP2/EREBP family is a class A gene for floral organ development (Nilsson et al. 2007), and its expression represents the collection of various early signals. In this study, eight of the 87 genes with the AP2 domain in hybrid larch in this period were highly similar to *AP2*, and one gene (c82659.graph_c0) showed a significant difference between the multiseeded type and the less-seeded type, indicating that this gene is closely related to hybrid larch reproduction and is likely to be the initiator of reproductive organ development (Yant et al. 2010). In addition, most genes in the RAV subfamily also regulate flowering. For example, *AtRAV1* regulates circadian rhythm (Mittal et al. 2015), *AtTEM1* and *AtTEM2* can inhibit flowering (Osnato et al. 2012), and *AtCHO* regulates the transformation of leaf structures (which is considered to be the basis of flower development) (Tsuwamoto et al. 2010). Therefore, c93691.graph_ c0, which is classified into this subfamily and differs across the two groups of materials, may be involved in the regulation of hybrid larch reproduction.

There are few studies on other subfamilies of AP2/EREBP genes involved in flowering. Most of the functions of these subfamilies are related to the biosynthesis of a variety of plant hormones (such as *AtERF11*, *AtERF022* and *AtDREB2C*, which are involved in the biosynthesis of GA, CK and ABA, respectively) (Je et al. 2014, Nowak et al. 2015, Zhou et al. 2016), hormone signal transduction, such as *AtSAN5*, which is involved in ABA signal transduction (Zhou et al. 2021), and the regulation of stress resistance (Scarpeci et al. 2017). However, in recent years, some studies have shown that these subfamilies may be involved in flowering regulation. For example, *AtREM16*, a TF and member of the ERF-B3 subfamily, can accelerate the flowering of *Arabidopsis* by upregulating *CO/FT/LFY* and *SOC1* (Yu et al. 2020). *AtERF1* within this subfamily can directly inhibit FT to delay flowering (Chen et al. 2021). *CmERF110* of the ERF-B4 subfamily can interact with *CmFLK* to regulate the biological clock in response to circadian rhythm to accelerate flowering (Huang et al. 2022). These results show that the AP2/EREBP TF family has great potential in the study of plant flowering. The 23 DEGs from the AP2/EREBP family identified in this study can be used as candidate genes for the study of reproductive development induction in hybrid larch in the future.

### Role of the WRKY and Myb DNA-binding gene families

In this study, TFs were enriched in the WRKY and MYB families in addition to the AP2 family. WRKY and MYB are two large gene families that are involved in almost every aspect of plant growth and development (Dubos et al. 2010, Rushton et al. 2010); thus, it was no surprise that they were involved in flowering. For example, *AtWRKY71* can accelerate flowering by directly activating *FT* and *LFY* (Li et al. 2016), and *CpWRKY71* found in sugar beet can also promote flowering *in Arabidopsis* (Huang et al. 2019)*. AtMYB106* of the MYB family negatively regulates flowering time by inhibiting the expression of *FT* (Hong et al. 2021)*. TaMYB72* in wheat promotes the flowering of rice (Zhang et al. 2016). Since only some of the members of these two families are known to be involved in the regulation of flowering, most of the WRKY and MYB family members we found during the flower induction period of hybrid larch are not the genes known to be involved in flowering. Therefore, this study did not conduct an in-depth analysis of whether WRKY and MYB genes in hybrid larch are involved in the regulation of reproductive development. However, due to the great evolutionary difference between gymnosperms and the model plant *Arabidopsis*, it is possible that these two families play a role in regulating the reproductive development of hybrid larch.

## Conclusions

According to the morphological observations, the floral induction of hybrid larch ended in mid-July and subsequently began organ development. The contents of GA3 and ABA in the buds of multiseeded plants were significantly higher than those of less-seeded plants, but JA and SA showed the opposite trend. The differentially expressed TF families at the end of floral induction in hybrid larch were mainly enriched in AP2, MYB DNA binding and WRKY domains. In the study period, 87 AP2/EREBP family genes classified into 13 subfamilies were found in hybrid larch. Among the 23 DEGs identified, 10 were upregulated in DHB and 13 were downregulated. These genes may play an important role in the floral induction of hybrid larch.

## Supplementary Material

Appendix_tpac111

## Data Availability

All high-throughput transcriptome data has been uploaded to CNCB database (cncb.ac.cn) under accession number: CRA007533.
