# Supplementary material for: Anatomical observation and transcriptome analysis of buds reveal the association between the AP2 gene family and reproductive induction in hybrid larch (Larix kaempferi × Larix olgensis)
Source: Tree Physiol. 2022 Sep 23;43(1):118–29. doi: 10.1093/treephys/tpac111 (PMC9833870; doi:10.1093/treephys/tpac111)
Supplement: Appendix_tpac111 [file appendix_tpac111.docx]

| **Table S1** Primers used for q-RT PCR in this study | | |
| --- | --- | --- |
| Gene number | Forward primer（5’-3’） | Reverse primer（5’-3’） |
| c82659.graph_c0 | AAGGCACAGATGGACAGGG | CTGCGGTCTTTTCGTCGTC |
| c76973.graph_c0 | TCGGCACTTTTGATACCGC | CTGCTATCGGGGTGAAGGG |
| c81611.graph_c0 | GAGATCGTGGGGGAAATGG | GATGCAGCATCATAGGCACG |
| c85050.graph_c0 | CCGCCTCCACTAGCTTTATC | ACGGTCATGCTGCTGCG |
| c85598.graph_c0 | GATTGTGCAAGCACTCGTTC | TGGTCAGGCATACTCATATTCG |
| c87422.graph_c2 | GAGCTTAACAGCCAGGTGG | ATGCAACCTTTCTTTGACCC |
| c88590.graph_c0 | TGGCTTGGTACTTTTGATACCG | TGCTACTCTGGTTGTCCCGC |
| c89534.graph_c0 | ATGACGCCGCATTGTATTG | GCATCCTTAATTTGCTGACG |
| c91214.graph_c0 | AAAGCAATGAATTCGCAGC | GATTTCCAGCTTGAACCCG |
| c93146.graph_c1 | AAGAGAAAGGAACTGGCCG | CCATACACGAGCTGCCTTC |

Appendix
